# Assessment of PI3K/mTOR/AKT Pathway Elements to Serve as Biomarkers and Therapeutic Targets in Penile Cancer

**DOI:** 10.3390/cancers13102323

**Published:** 2021-05-12

**Authors:** Anita Thomas, Sascha Reetz, Philipp Stenzel, Katrin Tagscherer, Wilfried Roth, Mario Schindeldecker, Martin Michaelis, Florian Rothweiler, Jindrich Cinatl, Jaroslav Cinatl, Robert Dotzauer, Olesya Vakhrusheva, Maarten Albersen, Stephan Macher-Goeppinger, Axel Haferkamp, Eva Juengel, Andreas Neisius, Igor Tsaur

**Affiliations:** 1 Department of Urology and Pediatric Urology, University Medicine Mainz, 55122 Mainz, Germany; anita.thomas@unimedizin-mainz.de (A.T.); sascha.reetz@unimedizin-mainz.de (S.R.); robert.dotzauer@unimedizin-mainz.de (R.D.); olesya.vakhrusheva@unimedizin-mainz.de (O.V.); sgoeppinger@gmail.com (S.M.-G.); axel.haferkamp@unimedizin-mainz.de (A.H.); eva.juengel@unimedizin-mainz.de (E.J.); a.neisius@bk-trier.de (A.N.); 2 Department of Pathology, University Medicine Mainz, 55122 Mainz, Germany; philipp.stenzel@unimedizin-mainz.de (P.S.); katrin.tagscherer@unimedizin-mainz.de (K.T.); wilfried.roth@unimedizin-mainz.de (W.R.); mario.schindeldecker@unimedizin-mainz.de (M.S.); 3 Industrial Biotechnology Centre and School of Biosciences, University of Kent, Canterbury CT2 7NJ, UK; M.Michaelis@kent.ac.uk; 4 Institute of Medical Virology, Goethe-University, 60596 Frankfurt am Main, Germany; f.rothweiler@kinderkrebsstiftung-frankfurt.de (F.R.); cinatl@em.uni-frankfurt.de (J.C.J.); ja.cinatl@kinderkrebsstiftung-frankfurt.de (J.C.); 5 Department of Urology, University Hospitals Leuven, 3000 Leuven, Belgium; maarten.albersen@uzleuven.be; 6 Department of Urology and Pediatric Urology, Krankenhaus der Barmherzigen Brüder Trier, 54292 Trier, Germany

**Keywords:** penile cancer, AKT, mTOR, cell lines, biomarker, targeted therapy

## Abstract

**Simple Summary:**

Penile cancer is a rare but aggressive malignancy characterized by rapid tumor growth as well as prompt metastasis in groin lymphatics. While localized diseases can be successfully cured by surgery in most cases, no truly effective treatment options have been established for metastatic diseases as of yet. In the current investigation, we assessed the value of selected members of the PI3K/mTOR/AKT pathway to serve as tumor markers or therapeutic targets for this disease. Higher expression of AKT was significantly more prevalent in high-grade tumors and independently predictive of the worse survival parameters, while increased expression of pmTOR was associated with an inferior prognosis as well. Treatment with the pan-AKT inhibitor capivasertib in PeCa cell lines induced significant reduction of cell viability and movement capacity. These findings might aid in the understanding of the molecular tumor background as well as development of novel treatment options for advanced penile cancer.

**Abstract:**

The PI3K/mTOR/AKT pathway might represent an intriguing option for treatment of penile cancer (PeCa). We aimed to assess whether members of this pathway might serve as biomarkers and targets for systemic therapy. Tissue of primary cancer from treatment-naïve PeCa patients was used for tissue microarray analysis. Immunohistochemical staining was performed with antibodies against AKT, pAKT, mTOR, pmTOR, pS6, pPRAS, p4EBP1, S6K1 and pp70S6K. Protein expression was correlated with clinicopathological characteristics as well as overall survival (OS), disease-specific survival (DSS), recurrence-free survival (RFS) and metastasis-free survival (MFS). AKT inhibition was tested in two primarily established, treatment-naïve PeCa cell lines by treatment with capivasertib and analysis of cell viability and chemotaxis. A total of 76 patients surgically treated for invasive PeCa were included. Higher expression of AKT was significantly more prevalent in high-grade tumors and predictive of DSS and OS in the Kaplan–Meier analysis, and an independent predictor of worse OS and DSS in the multivariate regression analysis. Treatment with pan-AKT inhibitor capivasertib in PeCa cell lines induced a significant downregulation of both total AKT and pAKT as well as decreased cell viability and chemotaxis. Selected protein candidates of the mTOR/AKT signaling pathway demonstrate association with histological and survival parameters of PeCa patients, whereas AKT appears to be the most promising one.

## 1. Introduction

Penile cancer (PeCa) is a rare but aggressive malignancy characterized by rapid tumor growth as well as prompt lymphatic metastasis [1]. In a localized disease, cure can be achieved in most patients. However, despite the increasing knowledge of molecular carcinogenesis and utilization of different systemic agents in clinical trials in the last decade, the prognosis still remains extremely poor for metastatic PeCa [2]. After failing the first-line, cisplatin-based chemotherapy, providing a progression-free survival of 20 weeks and response rate of 30%, overall survival falls short of 6 months [3]. Further scientific efforts targeted at profound deciphering of the molecular driver machinery in PeCa are ultimately warranted in order to pave the way for the development of more effective and individualized systemic protocols.

In this context, the preliminary data suggest that the PI3K/mTOR/AKT pathway might represent an intriguing option for the targeted treatment of PeCa. Phosphoinositide 3 kinase (PI3K) and its downstream mediator serine/threonine kinase AKT and mechanistic target of rapamycin (mTOR) constitute the core components of this signaling cascade, critically regulating such essential cellular processes as cell cycle progression and cell survival [4]. Importantly, activation of PI3K can be initiated by several mechanisms involving receptor tyrosine kinases such as the epidermal growth factor receptor (EGFR) and insulin-like growth factor-1 receptor (IGF-1R), cell adhesion molecules such as integrins, G-protein coupling receptors (GPCR) or oncogenes, such as Ras [5]. Upon activation, PI3K phosphorylates phosphatidylinositol-4,5-bisphosphate (PIP2) to phosphatidylinositol-3,4,5-trisphosphate (PIP3), which in turn activates AKT. At the same time, negative regulation of AKT can be achieved by dephosphorylation of PIP3 to PIP2 by the phosphatase and tensin homolog (PTEN) [4].

Alterations of proteins involved in PI3K/AKT/mTOR signaling are known to be involved in tumorigenesis of a plethora of malignancies. Activating mutations in the PIK3CA gene, which encodes the p110 catalytic subunit of PI3K, have been described in 36% of uterine endometrioid, 32% of colorectal and 25%–40% of breast cancer, resulting in the increased expression rate of total and phosphorylated AKT (pAKT) [6,7,8,9,10]. Moreover, overexpressed pAKT was detected in endometrial, prostate as well as pancreatic cancer [11,12]. For PeCa, scant evidence exists on the diagnostic and prognostic value of the molecular elements of the PI3K/AKT/mTOR signaling pathway thus far. In this study, we aimed at assessing the key players of this pathway for their potential to serve as tissue tumor markers and potential targets for systemic therapy.

## 2. Materials and Methods

### 2.1. Study Patients

A tissue microarray (TMA) consisting of PeCa tissue was used for this study. Tissue samples were provided by the tissue bank of the University Medical Center Mainz in accordance with the regulations of the tissue bank. All PeCa cases were collected from surgically treated patients with invasive PeCa from 1980 to 2018 at our institution and were confirmed blinded to the patient outcomes by experienced genitourinary pathologists. Disease management adhered to the currently valid European Association of Urology (EAU) Guidelines on Penile Cancer. All tumors were graded and staged according to the currently valid World Health Organization (WHO) and TNM classifications. A panel of clinical and histopathological parameters, including age at diagnosis, comorbidities, primary tumor surgery, tumor grade, pathologic T-stage, N-stage, status of infection with human papillomavirus (HPV), disease recurrence and its site, subsequent therapy and cause of death were collected for correlation with marker expression. Recurrence was classified as local (recurrent tumor on the penis), regional (recurrent disease in the inguinal or pelvic lymph nodes), or distant (remote metastasis), in adherence with a commonly used classification [13]. In the case of regional and distant recurrence in the same patient, it was classified as distant due to higher prognostic relevance. The follow-up protocol was defined as time from initial surgery until the last follow-up or death.

All patients with available formalin-fixed paraffin-embedded PeCA tissue samples were included in the TMA constructed as previously described [14]. First, representative tissue blocks were selected as donor blocks. From each donor block, sections were prepared and stained with H&E. Subsequently, from each PeCa tissue sample, a morphologically representative region was chosen. Two cylindrical core tissue specimens per tumor block (diameter, 1 mm) from these regions were punched and arrayed into the recipient paraffin block by usage of a semiautomatic system Tissue Arrayer MTA-1 (AlphaMetrix, Chicago, IL, USA).

### 2.2. Immunohistochemical Staining

The TMA slides were dewaxed and washed. Prior to immunohistochemical staining, a citrate buffer with pH 6 (Dako EnVision Flex Target Retrieval Solution low pH; DM829; lot 20048823, Agilent Technologies, Santa Clara, CA, USA) was used for heat-induced epitope retrieval. Staining was performed by using an automated staining system (DAKO Autostainer^plus^) and Envision Flex Kit (Dako EnVision TM FLEX HRP/Dab; K 8010), according to manufacturers’ instructions. The following antibodies were used: p4EBP1 (T37/46) (Cell Signaling Technology, Danvers, MA, USA, #2855), pp70S6K (T389) (Cell Signaling Technology, #9206), pS6 (S235/236) (Cell Signaling Technology, #4858), pPRAS (T246) (Cell Signaling Technology, #2997), pmTOR (Cell Signaling Technology, #2976), panAKT (Cell Signaling Technology, #4691), S6K1 (Abcam, Cambridge, UK, # ab32359); all incubated for 30 min and mTOR (Cell Signaling Technology, #2983), and pAKT (S473) (Dako, #S473); both were incubated for 60 min. An avidin-biotin-complex peroxidase technique, using aminoethylcarbazole for visualization and hematoxylin for counterstaining, was applied. Positive and negative controls were performed. pRaptor (Ser792) (Cell Signaling Technology, #2083) was very weakly expressed in the PeCa tissue and therefore was excluded from further analysis.

### 2.3. p16^INK4a^ Staining

Prior to immunohistochemical staining of p16 EDTA, buffer pH 9 (Dako EnVision Flex Target Retrieval Solution high pH; K 8004; lot 20062462) was used for heat-induced epitope retrieval. Staining was performed by using an automated staining system (DAKO Autostainerplus) and Envision Flex Kit (Dako EnVision TM FLEX HRP/Dab; K 8010) according to manufacturers’ instructions. The following antibodies were used: p16 CINtec Histology Kit (Roche, Basel, Switzerland, #E6H4) for 30 min. An avidin-biotin-complex peroxidase technique, using aminoethylcarbazole for visualization and hematoxylin for counterstaining, was used.

### 2.4. Staining Evaluation

**Figure 1:**
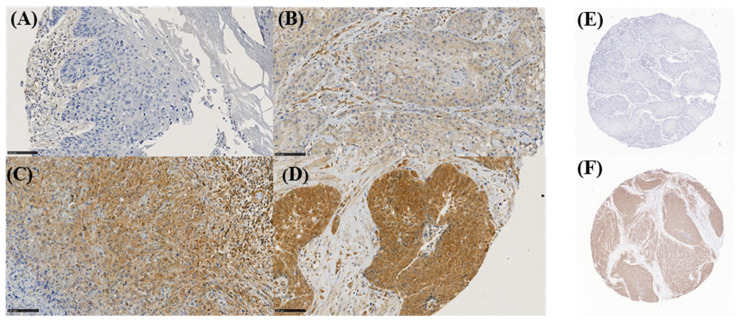
Immunohistochemical PeCa tissue sections stained with AKT antibody representing intensity score grades in 0 (**A**), 1 (**B**), 2 (**C**) and 3 (**D**), scale bar = 100 µm. p16INK4a staining was graded in negative (**E**) and positive (**F**).

The immunohistochemical semi-quantitative assessment of tumor markers expression was determined by the staining intensity and quantity of immunoreactive tumor cells. The intensity score was graded as 0 (negative), 1 (low), 2 (medium) and 3 (high) (Figure 1A–D).

The quantity score (percentage of positive cells) ranged from 0 (negative), 1 (positivity in <25%), 2 (positivity in <50%) to 3 (positivity in >50%). The final immunoreactive score (IRS) was calculated by the product of staining intensity and quantity scores, ranging from 0 to 9 [15]. The optimal cut-off values of IRS for discrimination between the groups with a “low” and “high” marker expression were determined with the Youden Index calculated on a receiver operating characteristic (ROC) curve based on patient survival data (Table S1) [16].

The immunohistochemical assessment of *p16^INK4a^* expression was classified as negative or positive (Figure 1E,F, respectively).

### 2.5. HPV DNA Detection

The total DNA was extracted from formalin-fixed and paraffin-embedded PeCa samples, using the standard kit (RSC DNA FFPE PLUS Custom Kit AX 4920, Promega, Madison, WI, USA) according to the manufacturers’ instructions. The presence of high-risk HPV was detected by PCT-based Sanger sequencing (CEQ 8000, Beckman Coulter, Brea, CA, USA), using the following primers and probes: Human Papilloma Virus E2 Gen: Primer 4XF, sequence 5′-GTAACACTACGCCTATAATACA-3′, Primer: 184559R, sequence 5′-CCTGTCCAATGCCAGGT-3′; Human Papilloma Virus E1 Gen: Primer 311635F, sequence 5′-ATAGSYATGTTAGATGATGCTACA-3′, Primer: 6Pack-R, sequence 5′-CACGTCCTTGAGAAAAAGGAT-3′; Major Capsid Protein L1-Region: Primer: MY11, sequence 5′-GCMCAGGGWCATAAYAATGG-3′, 5′-CGTCCMARRGGAWACTGATC-3′, Primer: MY09 sequence 5′-GATCAGTWTCCYYTKGGACG-3′. Genome Lab (GeXP Genetic Analysis System, Beckman Coulter, Brea, CA, USA) was used for interpretation.

### 2.6. Cell Culture

Human penile cancer cell lines UKF-PeC-3 and UKF-PeC-4 were established from patients with PeCa as described previously [17,18]. In brief, samples of diagnosed PeCa tissue were cut into small pieces and treated twice with trypsin (0.2%) for 30 min. Trypsin was inactivated by 10% FBS and cells were seeded in EpiMedium (ScienCell) until cell adhesion. The media were changed at five day intervals. The UKF-PeC-3 cell line was derived from a tumor with a pT3 pN0 L0 G2 R0 histopathological PeCa and was positive for HPV DNA subtype 16. UKF-PeC-4 was isolated from a tumor with pT2 pN1 (1/27) L0 V0 PN0 G2 R0 histopathological PeCa and was negative for hrHPV DNA. UKF-PeC-3 cells were cultured in IMDM supplemented with 10% FCS, 1% glutamax (Gibco, Thermo Fisher Scientific, Darmstadt, Germany), and 1% Anti/Anti (Gibco, Thermo Fisher Scientific, Darmstadt, Germany). UKF-PeC4 was grown in Epithelial Cell Medium (ScienCell Research Laboratories, Carlsbad) according to the manufacture’s manual at 37 °C in a humidified atmosphere with 5% CO2. Mycoplasma contamination was routinely examined. Cell viability was assessed by Trypan blue staining (Gibco/Invitrogen, Darmstadt, Germany). Cell lines were authenticated and validated (2021) using the primary tumor tissue by means of STR (Short Tandem Repeats) profiling (Institute of Legal Medicine, Goethe-University Frankfurt, Frankfurt, Germany).

### 2.7. Therapeutic Agent

The AKT inhibitor, capivasertib (AZD5363, AstraZeneca, UK), was used for in vitro studies. This compound is a novel synthetic AKT inhibitor that is orally bioavailable and was previously used in clinical trials [19,20]. For in vitro studies, capivasertib was dissolved in dimethyl sulfoxide (DMSO) at 10 mmol/L stock solution and stored at −20 °C.

### 2.8. Measurement of Cell Viability

Cell viability was tested by using the 3-(4,5-dimethylthiazol-2-yl)-2,5-diphenyltetrazolium bromide (MTT) dye. UKF-PeC-3 and UKF-PeC-4 cells were seeded into 96-well tissue culture plate and treated with capivasertib at concentrations ranging between 0.3125 and 80 µg/mL. After 24, 48 and 72 h incubation at 37 °C in an atmosphere containing 5% CO_2_, MTT (0.5 mg/mL) was added for 4 h. Subsequently, cells were lysed in a buffer (10% SDS in 0.01 M HCl) and the plates were incubated overnight at 37 °C with 5% CO_2_. Absorbance was detected at 570 nm by a multimode microplate-reader (Tecan, Spark 10 M, Crailsheim, Germany). To demonstrate dose–response kinetics, the mean cell number after 24 h incubation was set to 100%. Each experiment was done in triplicate.

### 2.9. Chemotatic Activity of PeCa Cell Lines

Serum-induced chemotactic activity was investigated using 24-well Transwell chambers with inserts (Corning, Glendale, Arizona, USA). UKF-PeC-3 and UKF-PeC-4 cell lines, pre-stained with CellTracker Green CMFDA (Thermo Fisher Scientific, Darmstadt, Germany), were incubated in the absence or the presence of 20 mol/L capivasertib for 72 h. PeCa tumor cells (6 × 10^5^/mL) were placed in the inserts (Corning, Glendale, AZ, USA) in a cell culture medium containing 10% serum. The lower chamber contained cell culture medium with 30% serum (Gibco, Thermo Fisher Scientific, Darmstadt, Germany). After incubation of 24 h at 37 °C, the lower surface and insert of the Transwell membrane were washed and gently wiped with a cotton swab to remove not migrated cells. Cells that had migrated to the lower surface of the membrane were assessed and counted by Sapphire Biomolecular Imager (Azure Biosystems, Inc., Dublin, CA, USA). Each experiment was done in triplicate.

### 2.10. Western Blot

To validate the expression blockade of AKT and pAKT under capivasertib treatment, a Western blot analysis was performed. PeCa cell lines were incubated in the absence or the presence of 20 mol/L capivasertib for 72 h. Tumor cell lysates (50 µg; protein concentration was quantified by BCA assay) were loaded onto a 7% polyacrylamide gel and were electrophoresed for 10 min at 80 V and for 60–90 min at 120 V. The proteins were then transferred to nitrocellulose membranes for 1 h at 100 V. After blocking with non-fat dry milk for 1 h, the membranes were incubated overnight at 4 °C with monoclonal antibodies directed against AKT (clone 55/PKBa/Akt, mouse immunoglobulin IgG1, dilution 1:1000, Becton Dickinson, Franklin Lakes, NJ, USA) and pAKT ((Ser473) (D9E); rabbit immunoglobulin IgG1; dilution 1:500; Cell Signaling Technology, Danvers, MA, USA). Horseradish peroxidase-conjugated goat-anti-mouse IgG (AKT) and goat-anti-rabbit IgG (pAKT) (IgG, both: dilution 1:1000, Dako, Glosturp, Denmark) served as the secondary antibody (30 min incubation at room temperature).

The membranes were shortly incubated with ECL detection reagent (AC2204, Azure Biosystems, Munich, Germany). Proteins were then visualized with a Sapphire Imager (Azure Biosystems, Munich, Germany). Coomassie staining served as an internal loading control. The ratio of protein intensity/whole protein intensity was calculated and expressed in percentage, related to untreated controls, set to 100%.

### 2.11. Statistical Analysis

Data were analyzed using the R software package (www.rproject.org; Version 3.6.0, accessed on 24 April 2021) and R Studio (Version 1.2.1335; www.rstudio.com, last access on 24 April 2021). For the count data, Fisher’s exact test (two-sided) was used. The Kaplan–Meier method was applied to calculate survival rates (overall (OS), recurrence-free (RFS), disease-specific (DSS) and metastasis-free survival (MFS)). Univariate survival data were tested for significance using the Mantel–Haenszel log-rank test. For multivariate analysis, the Cox proportional hazards regression model was used. Time-to-event variables were defined as the following: OS—time from the surgical resection to death due to any cause; DSS—time from the surgical resection to death caused by disease; RFS—time from the surgical resection to disease recurrence (local, regional or distant) or death from any cause; MFS—time from the surgical resection to first metastasis (regional or distant). Only metachronous events for recurrence or metastasis, defined as those occurring at least 3 months after surgery of the primary tumor, were included in MFS and RFS calculations. Correlation between the two parameters was evaluated by the analysis of Spearman’s coefficient.

All in vitro experiments were performed at least three times. Statistical significance was calculated with GraphPad Prism 7.0 (GraphPad Software Inc., San Diego, CA, USA): two-sided *t*-test (Western blot and chemotaxis) and two-way ANOVA test (MTT). Correction for multiple comparisons was done using the conservative Bonferroni method. Differences were considered statistically significant at a *p*-value ≤ 0.05.

## 3. Results

### 3.1. Clinicopathological Data

Between 1980 and 2018, we identified a total of 76 males with a cM0 PeCa with tissue from invasive primary tumors suitable for analysis (Table 1). The median age was 67 years (interquartile range, 31–86 years). Eight patients (10.5%) underwent circumcision, 3 patients (3.9%) underwent wide local tumor excision, 44 patients (57.9%) underwent partial penectomy and 21 patients (27.6%) underwent total penectomy as a primary treatment. Seventeen patients (22.4%) had high-grade primary tumors, 18 patients (23.7%) presented with a locally advanced disease and 14 patients (18.4%) had node-positive disease (pN1-3). Presence of a high-risk (hr)HPV was detected in 18 cases (23.7%; 16× HPV 16 and 2× HPV 59). p16^INK4a^ status was positive in 38 patients (50.0%). Overall, 18 tumors qualified as HPV-induced (concomitant HPV+/p16^INK4a^+ status). p16^INK4a^ immunopositivity was significantly associated with presence of HR-HPV DNA (*p* < 0.001; Fisher’s exact test). In total, the sensitivity of p16^INK4a^ staining for the presence of HR-HPV was 90% (specificity 62.26%, positive predictive value (PPV) 47.37%, negative predictive value (NPV) 94.29%).

### 3.2. Tumor Marker Expression and Associations

AKT expression was increased in 10 patients (13.2%; Table S2; representative image of different IRS scores—Figure 1), whereas its higher levels were significantly more prevalent in high-grade tumors (Figure S1). In contrast, it was independent of pT-stage, age at diagnosis, presence of lymph node metastasis or HPV infection.

Expression of pmTOR was elevated in 21 patients (27.6%; Table 2), while its higher values were less common in high-grade primary tumors (*p* = 0.029; Figure S1). Moreover, there was no impact of the primary pT-stage, age at diagnosis, presence of lymph node metastasis or HPV infection on the pmTOR expression. There were no significant associations between the level of expression of pmTOR or its unphosphorylated form mTOR with clinicopathological parameters (in particular, obesity and diabetes) in the univariate analysis (Figure S2).

Expression levels of mTOR, pAKT, pS6, p4EBP1, pp70S6K, s6k1 and pPRAS are depicted in Table S2. There were no significant associations between the level of expression of these proteins with clinicopathological parameters in the univariate analysis (Table S3). Moreover, positivity for hrHPV DNA was not associated with clinicopathological characteristics, prognosis and expression of marker candidates.

**Figure 2:**
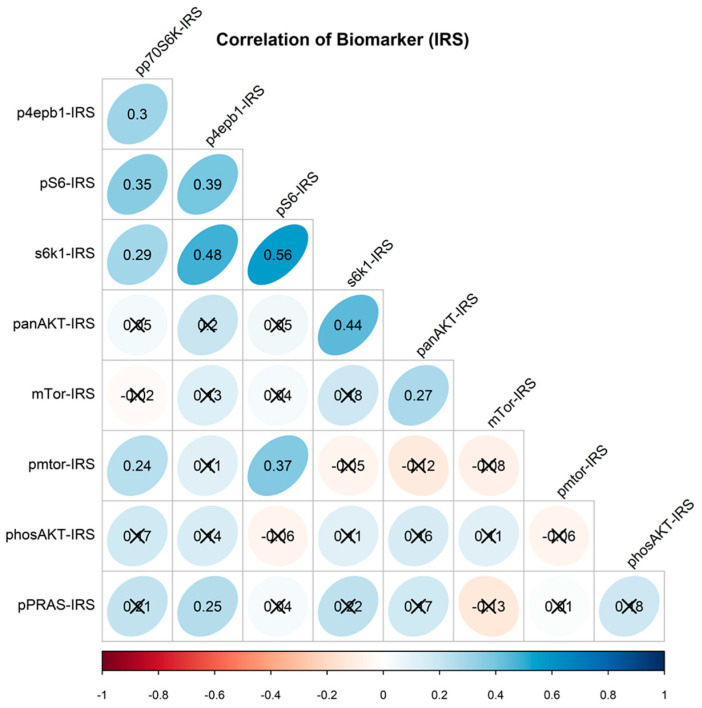
Correlogram depicting relationship between biomarkers. Correlation between two parameters was evaluated by the analysis of Spearman’s coefficient. Value of the respective association is delineated by the Spearman correlation coefficient (rs) in the oval. Blue ovals—positive rs, red ovals—negative rs. Color shade of the ovals represents the strength of the association (the darker—the stronger). Scored-out ovals—non-significant association.

Expression levels of mTOR as well as that of AKT did not correlate with that of its respective phosphorylated form pmTOR and pAKT. There was a respective positive association between the expression of s6k1 and AKT (*p* = 0.002), p4EBP1 (*p* = 0.001), and pS6 (*p* < 0.001). In addition, the expression of pS6 was respectively associated with pmTOR (*p* = 0.001), pp70S6K (*p* = 0.003) and p4EBP1 (*p* = 0.001) expression (Figure 2).

### 3.3. Follow-Up and Survival

During the median follow-up of 34.1 months for the total cohort, twenty-three patients (30.3%) died, including eight cancer-specific deaths (10.5%). In total, 18 patients (23.7%) developed recurrent disease comprising 8 patients (10.5%) with local recurrence, one patient (1.3%) with regional recurrence and 9 patients (11.8%) with distant recurrence. Median time to lymphatic recurrence after first surgical treatment was 7.6 months. Eleven patients (14.5%) were subjected to adjuvant chemotherapy. Five patients (6.6%) received palliative therapy, of which two patients (2.6%) were treated with chemotherapy alone, two patients with chemoradiotherapy and one patient (2.6%) received palliative radiation.

In the univariate COX regression analysis, lymph node metastasis was associated with a shortened DSS (95% confidence interval [CI], HR 28.12 (3.39–232.94; *p* = 0.002) (Figure S3). The univariate COX regression did not show statistically significant associations of clinical parameters with OS and RFS.

**Figure 3:**
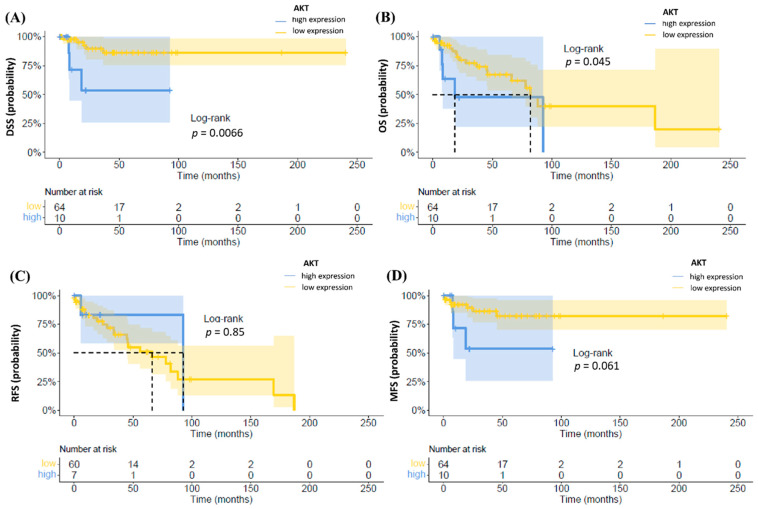
Kaplan–Meier plots of DSS (**A**), OS (**B**), RFS (**C**) and MFS (**D**) according to AKT. High expression of AKT was associated with a worse OS and DSS (*p* = 0.045 and *p* = 0.0066).

Upon Kaplan–Meier analysis, higher expression of AKT was associated with worse DSS and OS and a trend for inferior MFS as compared to the lower expression (*p* = 0.045, *p* = 0.007 and 0.061, respectively), while there was no difference in RFS between both groups (Figure 3).

**Figure 4:**
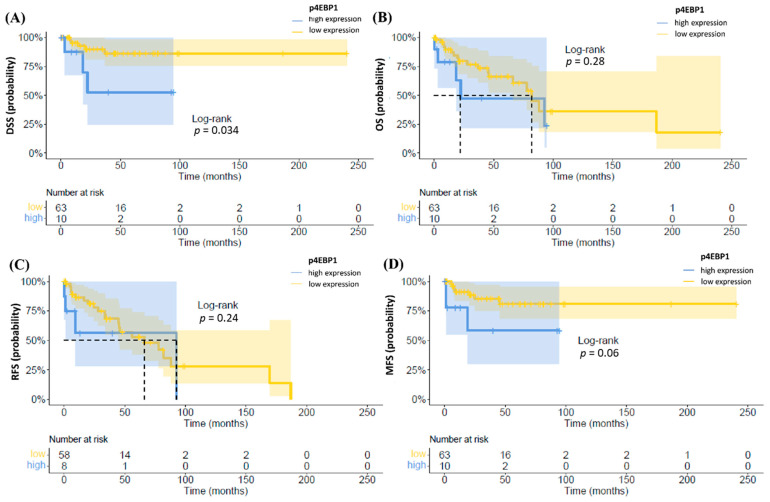
Kaplan–Meier plots of DSS (**A**), OS (**B**), RFS (**C**) and MFS (**D**) according to p4EBP1. High expression of p4EBP1 was associated with decreased DSS (*p* = 0.034).

In addition, higher levels of p4EBP1were significantly associated with an inferior DSS (*p* = 0.034), while there were no differences in OS and RFS (Figure 4). Additionally, a trend for unfavorable MFS associated with higher expression of p4EBP1 was observed (*p* = 0.06).

Upon Kaplan–Meier analysis, expression of pmTOR was not associated with OS, RFS and MFS, while a trend was observed for DSS (*p* = 0.064) (Figure S4).

In the multivariate COX regression analysis (Table 2), higher expression of AKT was an independent predictor of worse OS (HR 3.43; 95% CI, 1.22–9.68; *p* = 0.02) and DSS (HR 7.53; 95% CI, 1.12–50.5; *p* = 0.038). In addition, an increased expression of pmTOR (HR 9.95; 95% CI, 1.25–79.3; *p* = 0.03) was an independent predictor of worse DSS.

Regarding clinicopathological parameters, correlation of lymph node metastasis with inferior DSS and MFS was confirmed in the multivariate analysis (HR 36; 95% CI, 3.33–391; *p* = 0.003, HR 11.97; 95% CI, 2.70–53.5.12; *p* = 0.001, respectively) (Table 2). In addition, diabetes was an independent predictor of the worse (hazard ratio (HR) 3.86; 95% confidence interval (CI), 1.07–13.86; *p* = 0.038) as well as the presence of phimosis (hazard ratio (HR) 0.246; 95% confidence interval (CI), 0.07–0.89; *p* = 0.033) of the more favorable RFS.

### 3.4. Impact of AKT Blockade on Tumor Cell Growth

**Figure 5:**
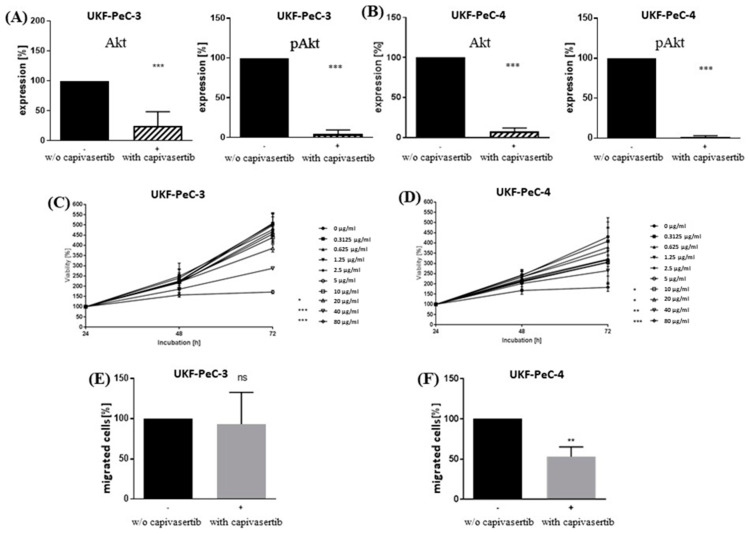
Molecular and functional effects of cell treatment with capivasertib. (**A**,**B**): Western blot analysis of AKT and pAKT after treatment with 20 mol/L capivasertib for 72 h for UKF-PeC-3 and UKF-PeC-4 cells, respectively. Cell number set to 100% after 24 h incubation. (**C**,**D**): Tumor cell growth of UKF-PeC-3 and UKF-PeC-4 cells after 24, 48, and 72 h exposure to capivasertib [0.3125–80 µg/mL], respectively. (**E**,**F**): Serum-induced chemotaxis of UKF-PeC-3 and UKF-PeC-4 cells after treatment with 20 mol/L capivasertib for 72 h, respectively. Cell number set to 100% after 24 h incubation. Error bars indicate standard deviation (SD). Significant difference to untreated control: * *p* ≤ 0.05, ** *p* < 0.01, *** *p* ≤ 0.001. *n* = 4.

In both PeCa cell lines, successful blocking of AKT and pAKT protein expression by capivasertib was verified by Western blot analysis (Figure 5A,B and Figures S5 and S6). In order to evaluate the possible effect of the AKT blockade on the functional behavior of PeCa cells, the established cell lines UKF-PeC-3 und UKF-PeC-4 were exposed to the pan-AKT inhibitor capivasertib in the range of 0.3125 to 80 µg/mL. A dose- and time-dependent growth inhibition was demonstrated in both cell lines, compared to their respective untreated controls (Figure 5C,D). The effect was hereby slightly stronger in UKF-PeCa-4 cells since a significant growth inhibition was first reached with 10 µg/mL (vs. 20 µg/mL in UKF-PeCa-3) after 72 h. Thus, for subsequent motility studies on both PeCa cell lines, a working concentration of 20 µg/mL capivasertib was used. No signs of toxicity were shown by the trypan blue exclusion test.

### 3.5. Modulation of Motility by AKT Inhibition

A trend for suppression of chemotaxis was observed in UKF-PeC-3 cells after 72 h capivasertib 20 µg/mL treatment (Figure 5E). At the same time, exposure to the compound decreased chemotactic movement by almost 50% in UKF-PeC-4 cells as compared to the untreated controls (Figure 5F).

## 4. Discussion

Extensive molecular characterization resulting in effective targeted therapy concepts has recently sounded the bell for precision medicine in a number of malignancies. Unfortunately, the advent of personalized treatment in PeCa as a rare disease is still neglectable, not least because of a limited funding and inadequate interest of stakeholders in preclinical research on the one hand and the scarcity of tumor models precluding molecular evidence-based clinical trials on the other hand [21]. In this context, the PI3K/mTOR/AKT pathway, a paramount signaling network steering various physiologic cellular processes, might offer new approaches for targeted therapy of PeCa. Importantly, a recent genomic study in PeCa revealed somatic missense mutations in the PIK3CA gene to be present in 9%, and they convey the protein with more activity, thus stimulating the PI3K/mTOR/AKT pathway [22]. Of note, different elements of this pathway were found to be activated in human cancers [23]. Consequently, its blockade has been successfully evaluated in preclinical and clinical oncologic settings at different levels, demonstrating an induction of tumor regression and survival advantage [23]. During the last decade, a number of inhibitors of this pathway have been approved for clinical application by the U.S. Food and Drug Administration (FDA) [24]. In PeCa, molecular rationale for targeting members of this pathway is still vague, necessitating further preclinical data prior to its possible transfer from lab to life.

Recently, Azizi and co-workers demonstrated that males with PeCa and a low expression of pAKT were at a higher risk of disease recurrence and those with a low expression of its downstream protein pS6 had an increased mortality risk in their TMA study with 57 samples [25]. Moreover, high expression of pAKT was associated with low-grade disease. Importantly, in our analysis, neither pAKT nor pS6 were associated with clinicopathological parameters or survival. Methodological issues of both differentially utilized immunohistochemical scoring methods (H-score vs. IRS) and defined cut-off values (quartile method vs. Youden index) for the classification of the low vs. high level of marker expression might have contributed to the discrepancy between these two investigations. Interestingly, in another TMA project on 112 invasive PeCa, Chaux et al. reported higher pAKT expression in high-grade PeCa, in contrast to the data of Azizi et al. [26]. It is noteworthy, however, that we observed more frequently increased values of total AKT in high-grade disease, while its enhanced expression was independently predictive of a poor OS and DSS, pointing to the involvement of the PI3K/mTOR/AKT pathway in tumor cell survival and disease progression. Interestingly, a contemporary investigation of 90 samples of oral squamous cell carcinoma revealed an upregulation of total AKT but not pAKT in metastatic and non-metastatic tumor patients as compared to controls [27]. Remarkably, Javle and collaborators revealed an independent predictive ability of AKT for OS as opposed to pAKT in cholangiocarcinoma, even if association between the expression of AKT and pAKT was noticed [28]. In our study, there was no correlation between the expression of both parameters, suggesting a presumably differential potential for testing as a tumor marker beyond statistical issues. Inverse to our results, enhanced expression of AKT correlated with improved survival in that assessment.

Interestingly, unlike OS, DSS and MFS (trend), the expression of AKT was not associated with RFS in the present study. This may be attributable to the fact that the given time-to-event variable RFS includes local recurrences besides regional and distant metastases, whereas local recurrence has presumably no negative impact on overall and metastasis-free survival in PeCa [29]. Notably, the direction of the association between AKT or pAKT expression and prognosis appears to vary depending on the malignancy. For instance, a correlation between AKT activation and poor prognosis was shown for prostate cancer and malignant melanoma [30,31]. At the same time, conflicting evidence exists for non-small cell lung cancer (NSCLC). Thus, Lu and colleagues provided evidence for the overexpression of pAKT being associated with metastasis and poor prognosis by processing 341 NSCLC samples [32]. On the contrary, Shah and co-workers reported on pAKT as a favorable prognostic factor despite being associated with a high tumor grade in the analysis of 82 NSCLC specimens [33]. Importantly, AKT1 overexpression and gene amplification were demonstrated to provide cisplatin resistance in lung cancer cells [34]. Suppression of AKT1 successfully rendered the cells sensitive for cisplatin in that study. In another analysis, treatment of colon cancer cells with cisplatin induced significant elevation of AKT, emphasizing its role in the development of chemoresistance in cancers [35].

Given the strongest association of the total AKT expression out of all investigated candidates with poor features of PeCa in the current analysis and evidence for its crucial role in adverse tumor behavior for other human cancers, we tested the potential of AKT inhibition to exert antitumor properties in our two primarily established PeCa cell lines UKF-PeC-3 and UKF-PeC-4. Treatment with the pan-AKT inhibitor capivasertib induced a significant downregulation of both total AKT and pAKT, associated with decreased cell viability. Concurrently, significantly reduced chemotaxis was demonstrated for UKF-PeC-4. Capivasertib was previously shown to inhibit all three isoforms of AKT (AKT1, AKT2 and AKT3) in enzyme assays and reduce phosphorylation of its downstream substrates P70S6K or PRAS40 and in vitro growth of a subset of various cancer cell lines and xenografts [36,37]. Even if a correlation between capivasertib sensitivity and the presence of activating PIK3CA mutations, PTEN loss or inactivating mutation, or HER2 amplification was observed, several cell lines without these aberrations turned out to be highly sensitive to this agent [36]. Of note, enhanced phosphorylation of AKT was demonstrated by the treatment of breast and prostate cancer cell lines with capivasertib as a consequence of the protein being held in a hyperphosphorylated but catalytically inactive form following compound binding [36]. It is noteworthy that in that study, cells were exposed to the drug for a period of 2 h. Therefore, the observed upregulation of pAKT obviously depicts a short-term feedback mechanism of counteracting its functional inhibition. In the investigation presented here, treatment of cells with the compound lasted for 72 h, inducing sustainable suppression or degradation of both AKT and pAKT.

In concert with the findings of other researchers, no association of the examined mTOR/AKT pathway elements with the presence of hrHPV infection was detected [26,38]. Interestingly, the extent of the reduction of cell viability by capivasertib in our assessment was comparable between the cell line generated from the hrHPV positive patient (UKF-PeC-3) compared to that of the hrHPV negative patient (UKF-PeC-4). This observation might point to the therapeutic potential of AKT inhibitors to effectively combat not only HPV-dependent cancers with a well-described activation of the PI3K/AKT/mTOR pathway, but also HPV negative ones, and is hypothesis-generating for the future evaluation [39].

Most recently, the efficacy of capivasertib was assessed in the nonrandomized phase 2 trial entitled NCI-MATCH, which enrolled patients with AKT1 E17K-mutated metastatic tumors of different origin that either had progressed after at least one line of standard systemic treatment or for which no effective therapy exists [40]. This aberration increases activity of AKT signaling by promoting localization of AKT to the plasma membrane through a PI3K-independent mechanism [41]. In these pretreated malignancies, the overall response rate was 28.6% and 6-month progression-free survival of 50%, but treatment was discontinued due to toxic side effects in 31% of participants, thus yielding a tolerable safety profile. In addition, a double-blind phase 2 trial called PAKT randomized women with metastatic triple negative breast cancer to receive paclitaxel with or without capivasertib [42]. In the overall population, the risk of death was decreased by 39%, whereas in the subset of females with PIK3CA/AKT1/PTEN-altered tumors, it was reduced by 70%, using combination treatment as compared to paclitaxel monotherapy. The rate of grade 3/4 adverse events in the combination arm was 54.4%. These encouraging data, corroborated by our in vitro analysis, pave the way to test the concept of AKT inhibition in PeCa as a mono or combination therapy. In particular, taking into account the role of AKT in cisplatin resistance in different cancers as well as the observed sensitization of cisplatin-sensitive and -resistant ovarian cancer cells by adding capivasertib, combining AKT inhibitors with cisplatin as the hub of the systemic therapy for metastasized PeCa presents an intriguing novel therapeutic option [34,35,43]. Whether the presence of activating genomic alterations of the PIK3CA/AKT1/PTEN pathway represents a clinically relevant predictive factor for selecting PeCa patients for AKT inhibition has to be further elucidated. From the clinical point of view, several indications for the use of AKT inhibitors merit future scientific efforts in PeCa. Successful generation of our cell lines paves the way for the establishment of chemotherapy- and other drug-resistant sublines in which molecular concepts of overcoming resistance, including AKT blockade, can be assessed, aiming at identification of efficacious second-line treatment options after failure of the primary systemic treatment. Moreover, utilization of AKT inhibitors in neoadjuvant, adjuvant, topical cytoreductive or, in case of superficial lesions, curative indications is an appealing area of ongoing translational research.

Previously, Ferrandiz-Puildo and co-authors reported the overexpression of pmTOR to be associated with lymph node-positive disease and higher pT stages as well as that of p4EBP1 with lower pT stages in their TMA study on 67 men with invasive PeCa [44]. Surprisingly, in our investigation, augmented expression of pmTOR correlated with low-grade PeCa and was, at the same time, predictive of unfavorable DSS. At first glance, this result appears counterintuitive. However, higher tumor grade was not independently predictive of survival in the current analysis. In concert with our findings, several other groups also reported no association between the tumor grade and cancer progression or prognosis in PeCa [45,46,47,48]. Apart from a possible statistical bias related to the limited sample size of the study cohort, one explanation might be that mTOR activation in low-grade PeCa could accelerate translational activity and oxygen consumption as it was previously assumed for prostate cancer [49]. During transition from well to poorly differentiated disease, evolving hypoxia has the potential to suppress mTOR signaling, leading to the downregulation of pmTOR in high-grade tumors [50]. While tumor grade has no impact on prognosis in this investigation, the detrimental influence of mTOR activation on DSS turns out to be apparent in the multivariate analysis. Since no evidence exists on this issue in PeCa, research on hypoxia-associated aspects of this disease is warranted. In addition, it would be intriguing to assess the value of mTORC1 and mTORC2 in PeCa since the current project focused on total mTOR. Of note, association of the overexpression of pmTOR with mostly adverse cancer features were reported in other squamous cell carcinomas as well [27,51]. As previously reported, mTOR regulates lipid and glucose metabolism, thus playing a major role in metabolic diseases, such as obesity and diabetes mellitus type 2 [52]. Interestingly, neither obesity nor diabetes were associated with higher or lower mTOR/pmTOR expression in our studies.

In addition, increased content of p4EBP1 indicated a shorter DSS on the log-rank test, while a trend was observed for this association in the multivariable model (*p* = 0.09) in the current study. Since evidence on the potential value of this candidate to serve as a tumor marker or therapeutic target is scant (not only in PeCa), further research in this area is welcome.

Due to the rarity of the disease, the generalization of the findings of our study is hampered by the sample size limiting the stability of the statistical models as well as by the number of investigated cell lines. Additionally, since lymphovascular invasion and, hence, classification of pT1 stage in pT1a and pT1b was first available after 2010, limited information in these categories as observed in several other studies precluded their robust incorporation in our statistical models [53,54]. Nonetheless, we hope to have put a relevant piece to the scientific puzzle of the molecular background of PeCa, yielding hypothesis-generating results, emboldening future scientific efforts.

## 5. Conclusions

A number of proteins of the mTOR/AKT signaling pathway demonstrate association with the histological and survival parameters of patients with PSCC, and AKT appears to be the most promising one. This is further corroborated by in vitro experiments.

## Figures and Tables

**Table 1:** Clinical baseline characteristics of the study population.

Clinical Characteristics	Overall (*n* = 76)
Age at diagnosis	
Mean (SD)	64.1 (11.9)
Median [Min, Max]	67.0 [31.0, 86.0]
≤65	36 (47.4%)
>65	40 (52.6%)
Primary tumor surgery	
Circumcision	8 (10.5%)
Tumor excision	3 (3.9%)
Partial penectomy	44 (57.9%)
Total penectomy	21 (27.6%)
Primary tumor grade	
Low (G1/G2)	59 (77.6%)
High (G3/G4)	17 (22.4%)
Pathologic T stage	
pT1	32 (42.1%)
pT2	26 (34.2%)
pT3	18 (23.7%)
Pathologic T1 subtypes	
pT1a	7 (21,9%)
pT1b	2 (6,2%)
Missing	23 (71,9%)
Lymphovascular invasion	
No	31 (40.8%)
Yes	18 (23.7%)
Missing	27 (35.5%)
HPV infection	
Negative	56 (73.7%)
Positive	18 (23.7%)
Missing	2 (2.6%)
p16^INK4a^ status	
Negative	35 (46.1%)
Positive	38 (50.0%)
Missing	3 (3.9%)
Pathologic N stage	
NX-0	62 (81.6%)
N1	4 (5.3%)
N2	7 (9.2%)
N3	3 (3.9%)
Recurrence status	
No	58 (76.3%)
Yes	18 (23.7%)
Recurrence location	
None	58 (76.3%)
Local	8 (10.5%)
Regional	1 (1.3%)
Distant	9 (11.8%)
Subsequent therapy	
None	60 (78.9%)
CTX	13 (17.1%)
Radiation	1 (1.3%)
CTX and Radiation	2 (2.6%)
Tumor-dependent death	
No	68 (89.5%)
Yes	8 (10.5%)

**Table 2:** Multivariate COX regression analysis of survival endpoints.

Characteristic	OS		DSS		RFS		MFS	
	HR (95% CI)	*p*	HR (95% CI)	*p*	HR (95% CI)	*p*	HR (95% CI)	*p*
Age at diagnosis ≤65 >65 Lymph node metastasis							1.00 (reference) 3.07 (0.72–13.02)	0.128
Negative			1.00 (reference)				1.00 (reference)	
Positive			36 (3.33–391)	0.003			11.97 (2.70–53.12)	0.001
Primary tumor								
pT1					1.00 (reference)			
pT2/pT3					0.318 (0.09–1.16)	0.0837		
Diabetes								
Negative					1.00 (reference)			
Positive					3.86 (1.07–13.86)	0.0384		
HTN								
Negative					1.00 (reference)			
Positive					0.337 (0.09–1.23)	0.0987		
COPD								
Negative					1.00 (reference)			
Positive					2.86 (0.73–11.27)	0.133		
Phimose								
Negative					1.00 (reference)			
Positive					0.246 (0.07–0.89)	0.0333		
TNM N stage								
Negative					1.00 (reference)			
Positive					0.287 (0.05–1.67)	0.164		
Grading								
G1/G2					0.43 (0.16–1.18)	0.102		
G3/G4					1.00 (reference)			
AKT								
Low expression	1.00 (reference)		1.00 (reference)					
High expression	3.43 (1.22–9.68)	0.0197	7.53 (1.12–50.5)	0.0377				
pmTOR								
Low expression			1.00 (reference)					
High expression			9.95 (1.25–79.3)	0.0301				
p4epb1								
Low expression			1.00 (reference)					
High expression			5.99 (0.779–46.1)	0.0855				
pPRAS								
Low expression					1.00 (reference)			
High expression					0.132 (0.01–1.62)	0.113		

## Data Availability

All data generated or analyzed during this study are included in this published article and its supplementary information files.

## Supplementary Materials

The following are available online at https://www.mdpi.com/article/10.3390/cancers13102323/s1. Figure S1: Dichotomized biomarker expressions of AKT and pmTOR correlated with clinical and histopathological data (grading, primary tumor, lymph node metastases, HPV infection and age). The Fischer’s exact test was used for statistical analysis. High expression of AKT (*p* = 0.044) and low expression of pmTOR (*p* = 0.029) were associated with high-grade primary tumors. Figure S2: Expressions of mTOR and pmTOR correlated with clinical data (diabetes and obesity). *Y*-axis: immunoreactive score (IRS). *X*-axis: yellow boxplots—low expression of the respective biomarker, blue boxplots—high expression of the respective biomarker. Box (represents the interquartile range (IQR)): lower line—quartile Q1 (25%-quantile); middle line—median; upper line—quartile Q3 (75%-quantile); whisker—separate values lying outside the IQR; circles—outliers. The Fischer’s exact test was used for statistical analysis. Figure S3: Univariate COX regression analysis: lymph node metastasis was associated with a shortened disease-specific survival (DSS) (*p* < 0.0001). Figure S4: Kaplan–Meier Plots of disease-specific survival (DSS) (A), overall survival (OS) (B), recurrence-free survival (RFS) (C) and metastasis-free survival (MFS) (D) according to pmTOR. Figure S5: Protein expression profile in UKF-PeC-3 and UKF-PeC-4 cells without/after treatment with capivasertib. Protein expression of AKT (a), corresponding Coomassie blue staining of total protein (b). Figure S6: Protein expression profile in UKF-PeC-3 and UKF-PeC-4 cells without/after treatment with capivasertib. Protein expression of pAKT (a), corresponding Coomassie blue staining of total protein (b). Table S1: Immunoreactive scores (IRS). IRS determined as cutoff levels (low and high) after graphically depicting the survival curves of each of the scores separately by receiver operating characteristic (ROC). Table S2: Immunohistochemical staining results with a respective group classification. Values in brackets: percentage from total number. Table S3: Clinicopathological parameters stratified by expression of the assessed marker candidates.
