# Supplementary material for: Assessment of PI3K/mTOR/AKT Pathway Elements to Serve as Biomarkers and Therapeutic Targets in Penile Cancer
Source: Cancers (Basel). 2021 May 12;13(10):2323. doi: 10.3390/cancers13102323 (PMC8151654; doi:10.3390/cancers13102323)
Supplement: Supplementary file 1 [file cancers-13-02323-s001.zip › Figures S1-S6 Table S1.S2.pdf]

cancers

MDPI

Article

Assessment of PI3K/mTOR/AKT Pathway Elements to Serve as Biomarkers and Therapeutic Targets in Penile Cancer

Anita Thomas, Sascha Reetz, Philipp Stenzel, Katrin Tagscherer, Wilfried Roth, Mario Schindeldecker, Martin Michaelis, Florian Rothweiler, Jindrich Cinatl Jr., Jaroslav Cinatl, Robert Dotzauer, Olesya Vakhrusheva, Maarten Albersen, Stephan Macher-Goeppinger, Axel Haferkamp, Eva Juengel, Andreas Neisius and Igor Tsaur

Supplementary Materials

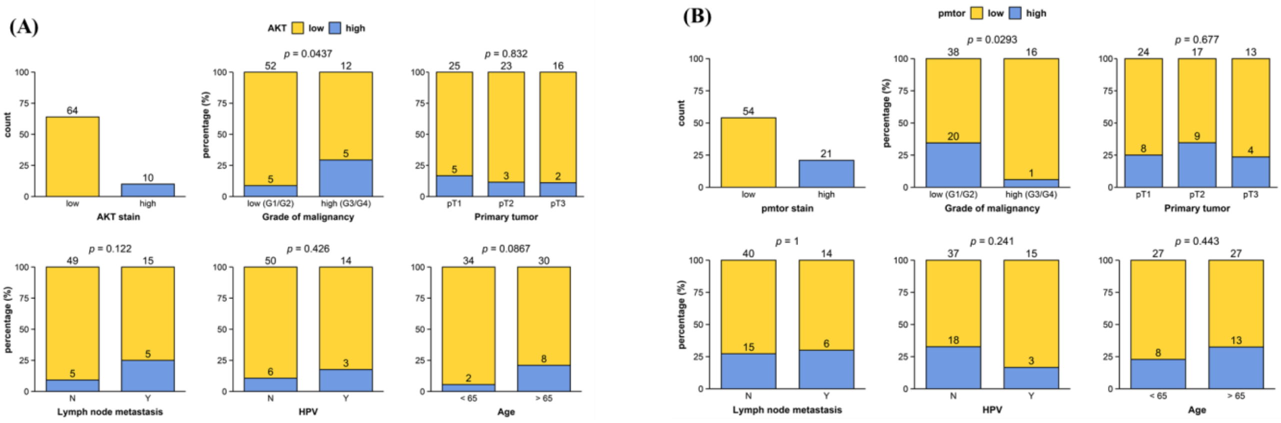

Figure S1. Dichotomized biomarker expressions of AKT (A) and pmTOR (B) correlated with clinical and histopathological data (grading, primary tumor, lymph node metastases, HPV infection and age). The Fischer's exact test was used for statistical analysis. High expression of AKT (p = 0.044) and low expression of pmTOR (p = 0.029) were associated with high-grade primary tumors

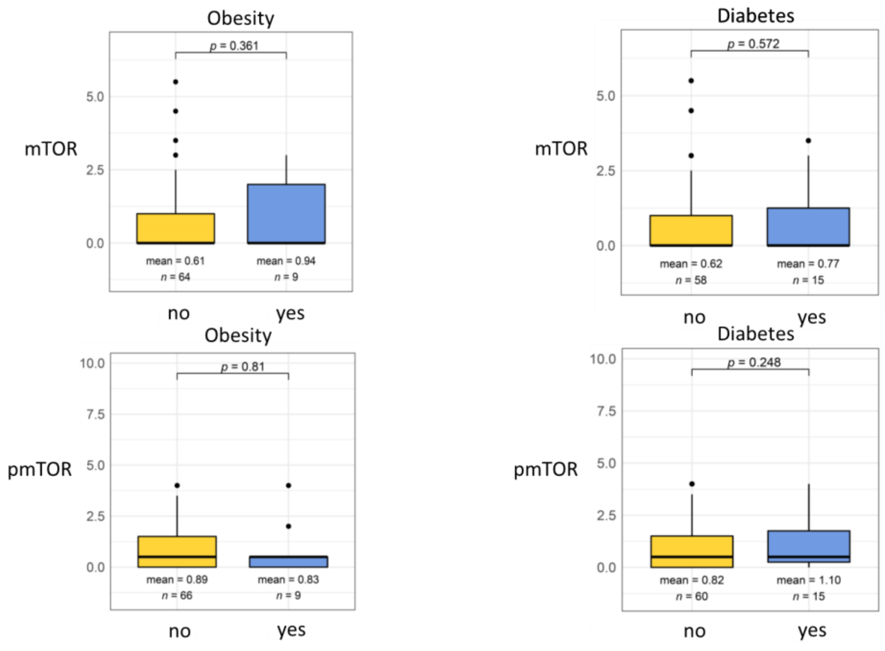

Figure S2. Expressions of mTOR and pmTOR correlated with clinical data (diabetes and obesity). *Y*-axis: immunoreactive score (IRS). *X*-axis: yellow boxplots—low expression of the respective biomarker, blue boxplots—high expression of the respective biomarker. Box (represents the interquartile range (IQR)): lower line—quartile Q1 (25%-quantile); middle line—median; upper line—quartile Q3 (75%-quantile); whisker—separate values lying outside the IQR; circles—outliers. The Fischer's exact test was used for statistical analysis.

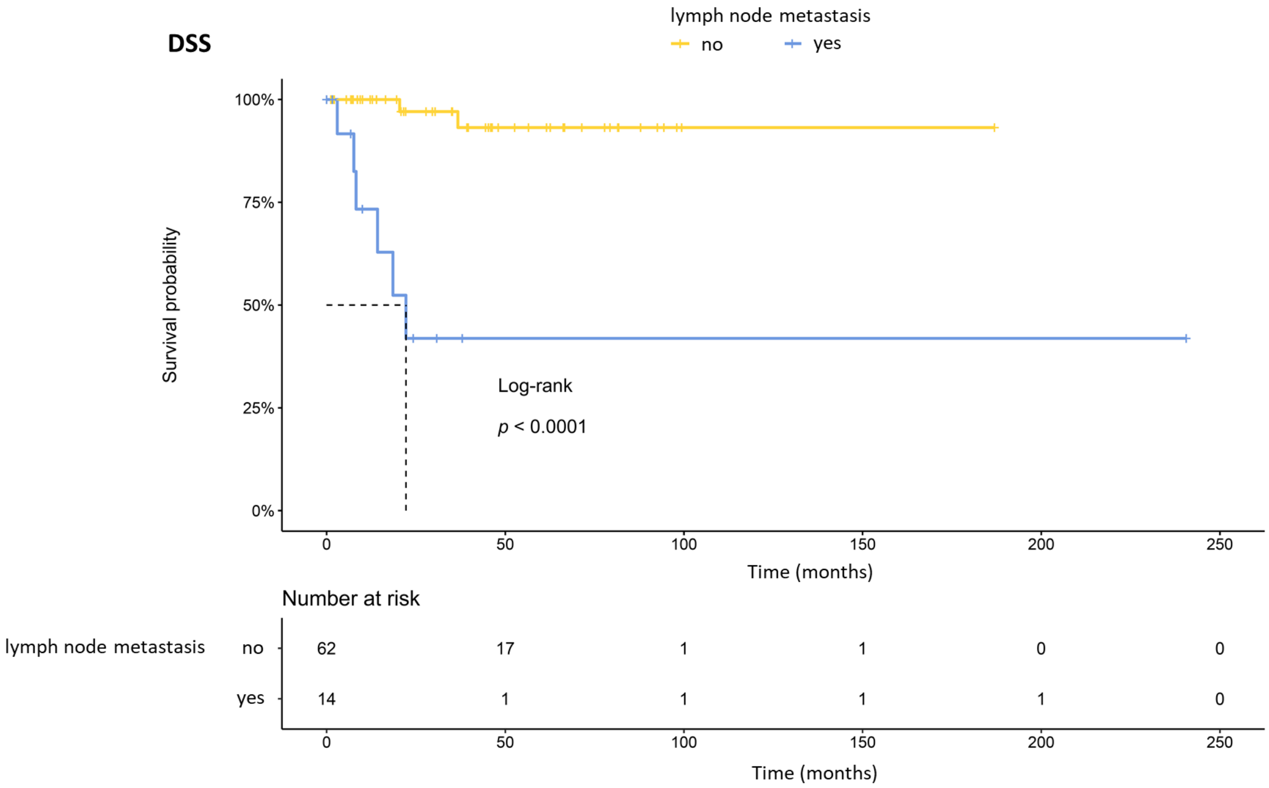

Figure S3. Univariate COX regression analysis: lymph node metastasis was associated with a shortened disease-specific survival (DSS) ($p < 0.0001$).

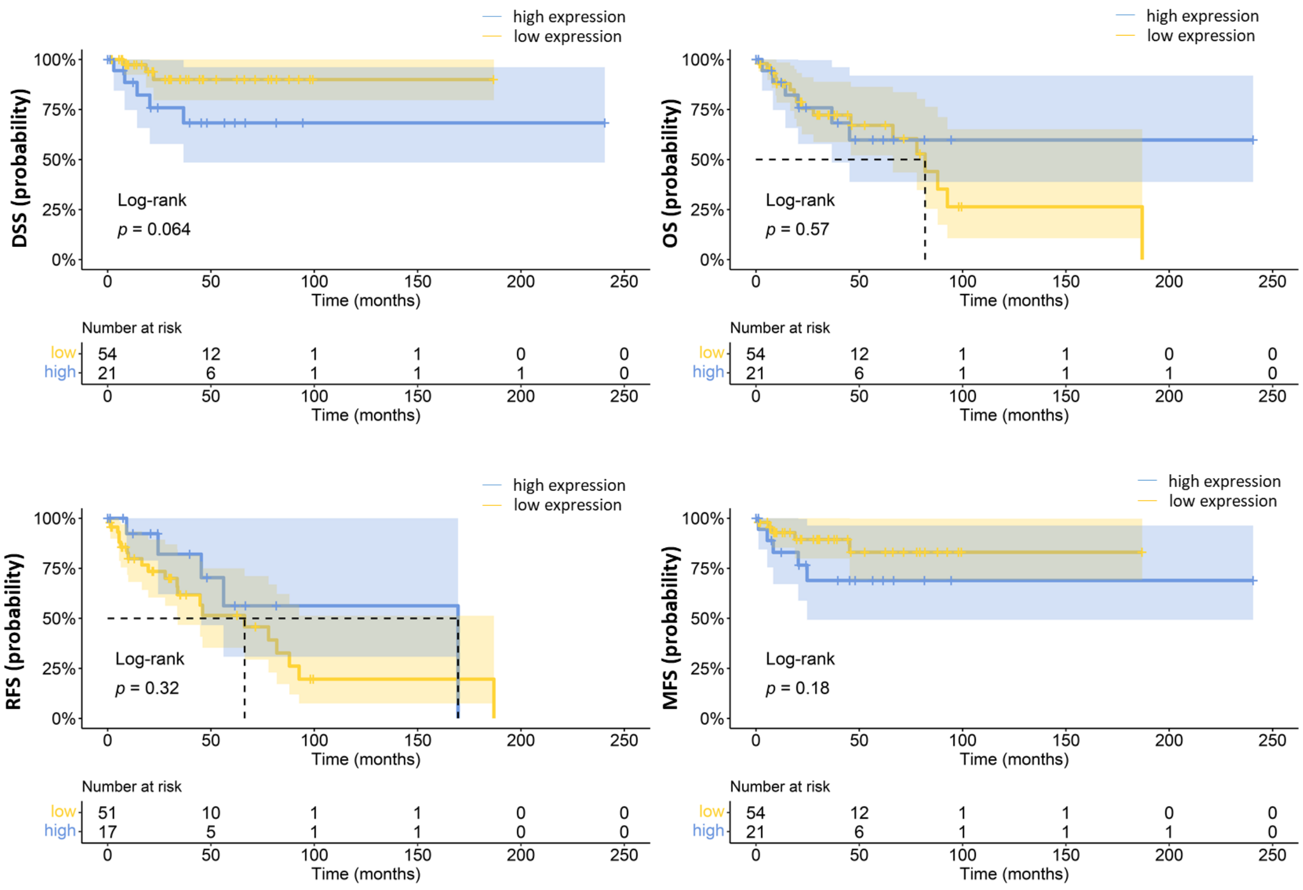

Figure S4. Kaplan–Meier Plots of disease-specific survival (DSS) (**A**), overall survival (OS) (**B**), recurrence-free survival (RFS) (**C**) and metastasis-free survival (MFS) (**D**) according to pmTOR expression.

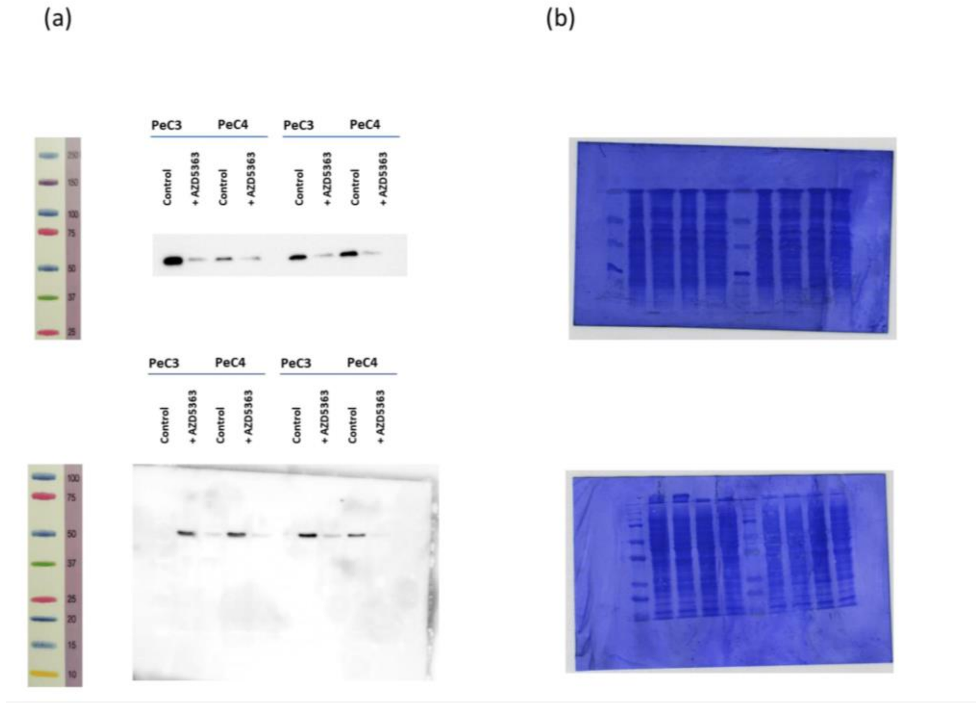

Figure S5. Protein expression profile in UKF-PeC-3 and UKF-PeC-4 cells without/after treatment with capivasertib. Protein expression of AKT (**a**), corresponding Coomassie blue staining of total protein (**b**).

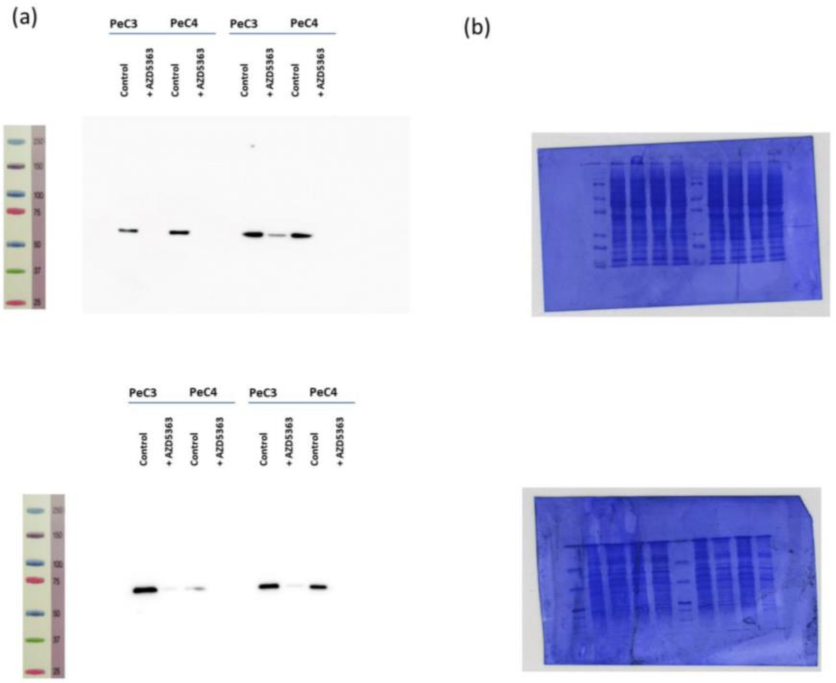

Figure S6. Protein expression profile in UKF-PeC-3 and UKF-PeC-4 cells without/after treatment with capivasertib. Protein expression of pAKT (**a**), corresponding Coomassie blue staining of total protein (**b**).

Table S1: Immunoreactive scores (IRS). IRS determined as cutoff levels (low and high) after graphically depicting the survival curves of each of the scores separately by receiver operating characteristic (ROC).

Biomarker	IRS cut-off
AKT	4,75
pAKT	0,75
pmTOR	1,25
pS6	1,25
p4EBP1	6,25
pPRAS	0,75
pp70S6K	0,25
mTOR	1,25
S6K1	0,25

Table S2. Immunohistochemical staining results with a respective group classification. Values in brackets: percentage from total number.

Biomarker	Overall (n = 76)
**panAKT**	
Low expression	64 (84.2%)
High expression	10 (13.2%)
Missing	2 (2.6%)
**pAKT**	
Low expression	20 (26.3%)

High expression	53 (69.7%)
Missing	3 (3.9%)
**mTor**	
Low expression	58 (76.3%)
High expression	15 (19.7%)
Missing	3 (3.9%)
**pmTor**	
Low expression	54 (71.1%)
High expression	21 (27.6%)
Missing	1 (1.3%)
**pS6**	
Low expression	23 (30.3%)
High expression	50 (65.8%)
Missing	3 (3.9%)
**p4epb1**	
Low expression	63 (82.9%)
High expression	10 (13.2%)
Missing	3 (3.9%)
**pp70S6K**	
Low expression	47 (61.8%)
High expression	26 (34.2%)
Missing	3 (3.9%)
**s6k1**	
Low expression	3 (3.9%)
High expression	44 (57.9%)
Missing	29 (38.2%)
**pPRAS**	
Low expression	71 (93.4%)
High expression	5 (6.6%)
